# Red Blood Cell Alloimmunization and Autoimmunization in Blood Transfusion-Dependent Sickle Cell Disease and *β*-Thalassemia Patients in Al-Ahsa Region, Saudi Arabia

**DOI:** 10.1155/2023/3239960

**Published:** 2023-04-28

**Authors:** Fahd A. Kuriri, Abdulrahman Ahmed, Fehaid Alanazi, Fahad Alhumud, Mohammed Ageeli Hakami, Osama Atiatalla Babiker Ahmed

**Affiliations:** ^1^Department of Clinical Laboratory Sciences, College of Applied Medical Sciences, Shaqra University, Shaqra, Riyadh Province, Saudi Arabia; ^2^Al-Ahsa Health Cluster, Al-Ahsa, Saudi Arabia; ^3^Department of Clinical Laboratory Sciences, College of Applied Medical Sciences-AlQurayaat, Jouf University, Sakaka, Saudi Arabia; ^4^Department of Clinical Laboratory Sciences, College of Applied Medical Sciences, Al-Quwayiyah, Shaqra University, Riyadh, Saudi Arabia

## Abstract

**Introduction:**

The risk of developing transfusion-related complications, especially alloimmunization, is an ongoing concern for transfusion-dependent patients. It is important to determine the rate of alloimmunization and autoimmunization in Al-Ahsa Region, Saudi Arabia, where sickle cell disease (SCD) and thalassemia incidence rates are the highest in Saudi Arabia.

**Methods:**

A cross-sectional study was conducted to review the transfusion history of patients with SCD and thalassemia at the King Fahad Hospital (KFH) in Al-Ahsa, Saudi Arabia. 364 transfusion-dependent patients were included in this study.

**Results:**

Alloimmunization rates in patients with SCD and thalassemia were 16.7% and 11.97%, respectively, while autoimmunization rates in patients with SCD and thalassemia were 5.3% and 0.7%, respectively. The most frequent alloantibodies among the study participants were against Kell, Rh blood group systems.

**Conclusion:**

Blood transfusion-related alloimmunization and autoimmunization compromise the proper management of chronically transfused patients. Ideally, extended matched phenotyping should be implemented to prevent alloimmunization and reduce the risk of developing blood transfusion-related alloantibodies.

## 1. Introduction

Prior to the discovery of the ABO blood groups by Karl Landsteiner in 1901, all blood was considered the same leading to critical blood transfusion side effects [1, 2]. These side effects were due to incompatibilities in ABO blood type between donors and recipients, but since 1901, transfusions became more safe as harmful incompatibilities were reduced. In transfusion medicine today, ABO blood group antigens remain critically important as they are the most immunogenic of all the blood group antigens [3]. One of the most common causes of morbidity in blood transfusion is blood type incompatibilities due to clerical error [1, 2]. Incompatible blood group antigens can induce immune reactions, known as alloimmunization, in patients who lack the corresponding antigens on their red blood cells (RBCs). Aside from the transfusion of antigen positive blood into antigen negative patients, alloimmunization can also occur during pregnancy [4]. In addition to alloimmunization, transfusions can also induce autoimmunization where transfusion-related autoantibodies are hypothesized to develop against the patient's own cells.

Sickle cell disease and *β*-thalassemia are common hemoglobinopathies in Saudi Arabia. According to a systematic review and meta-analysis of 18 studies, the prevalence of sickle cell disease in Saudi Arabia ranges from 0.9% to 4.2%, with the highest prevalence observed in the eastern and southern regions of the country [5]. *β*-thalassemia is also highly prevalent in Saudi Arabia, with a carrier rate of 3-4% in the general population [6]. In some areas, such as the Eastern Province, the prevalence of *β*-thalassemia is estimated to be as high as 15–20% [6].

Transfusion of RBCs is one of the most commonly used medical interventions in clinical settings accounting for more than 108 million units administered worldwide every year [7]. The major complication of regular blood transfusions, particularly in patients who are chronically transfused, is RBC alloimmunization [7]. Even though RBC alloimmunization mitigation strategies are implemented for chronically transfused patients, multitransfused patients are still at risk of developing alloantibodies [8]. Donor and recipient factors play a role in RBC alloimmunization. These factors range from characteristics of a particular blood group antigen to the recipient's ability to present the antigens to their immune system [9]. In addition to genetic factors, emerging data have highlighted the importance of environmental factors in the formation of RBC alloantibodies [10].

Recent studies investigating the prevalence and frequency of RBC antigens that cause hemolytic transfusion reactions in Saudi Arabia have focused on populations from Southwestern Saudi Arabia [11–13]. It is important to phenotype other populations in Saudi Arabia, particularly people from the Al-Ahsa Region where sickle cell disease (SCD) and *β*-thalassemia are endemic. These populations require regular blood transfusions to maintain healthy hemoglobin levels to improve oxygen-carrying capacity of blood and reduce the serious disease-associated complications [14, 15]. However, the development of alloantibodies associated with blood transfusions can negatively impact the health of these patients and complicate transfusion therapy [10]. This study was designed to identify the most common phenotypes associated with transfusion side effects among blood transfusion-dependent SCD and *β*-thalassemia patients from Al-Ahsa Region. Understanding these phenotypes will help lower the incidence of adverse transfusion outcomes by using extended phenotype matching to identify more compatible blood.

## 2. Subjects and Methods

### 2.1. Patients

This cross-sectional study was conducted in accordance with the code of conduct of research in Saudi Arabia and the 1964 Helsinki Declaration and its later amendments. This study was approved by the Research Ethical Committee of the Institutional Review Board of King Fahad Hospital in Al-Ahsa, Ministry of Health, Kingdom of Saudi Arabia (No. 33-EP-2022). The study was conducted over 5 months between May 2022 and October 2022 and included 362 patients, 284 transfusion-dependent *β*-thalassemia patients and 78 SCD patients. All patients received leukoreduced RBC transfusions.

Clinical and transfusion records of all patients were reviewed for demographic and clinical data including age, gender, age of initial transfusion therapy, transfusion frequency, total number of blood units transfused, and status of splenectomy. All data were collected with sociodemographic characteristics of participants included in Table 1.

### 2.2. Inclusion Criteria

Regardless of sex and age, only Saudi patients hospitalized for blood transfusions were included in this study.

### 2.3. Exclusion Criteria

Patients with a history of hematological malignancies were excluded from this study.

### 2.4. Transfusion Protocol

Transfusion policy adhered to international guidelines, including those established by the American Association of Blood Banks (AABB) and the Saudi Ministry of Health. To ensure the safety and efficacy of the transfusion process, a comprehensive screening was conducted to determine the presence of various blood group antigens, including A, B, O, AB, D, C, c, E, e, and K. The ID system gel cards (Bio-Rad, Dreieich, Germany) were utilized for antigen testing, while DiaClon ABO/D + reverse grouping and DiaClon Rh subgroups + K gel cards (Bio-Rad, Dreieich, Germany) were used for blood grouping and subtyping. Antibody screening and identification were also performed to detect the presence of any alloantibodies that may react with the donor's blood. Also, each sample underwent a direct antiglobulin test with a polyspecific antihuman globulin reagent (anti-IgG and anti-C3d) using the gel technique with a LISS/Coombs card (DiaMed GmbH, Switzerland), according to the method of Obaid et al. [16].

### 2.5. Statistical Analysis

A nonprobability sampling technique was utilized in this study. Sample size was calculated using a 95% confidence interval and 5% margin of error to include 385 participants. Frequencies of phenotypes in the Al-Ahsa population were compared with phenotype frequencies in other ethnicities by performing a chi-squared test. *P* values <0.05 and <0.01 indicated significant and highly significant differences, respectively.

## 3. Results

The rates of *β*-thalassemia and SCD alloimmunization in the 362 transfusion-dependent participants sampled in this study are presented in Table 2. Of the 362 transfusion-dependent participants, 284 (78.5%) were transfusion-dependent *β*-thalassemia patients and 78 (21.5%) were SCD patients. Of the *β*-thalassemia patients, 34 (11.8%) developed alloantibodies including 12 males (35%) and 22 females (65%). Of the SCD patients, 13 (16.7%) developed alloantibodies including 7 males (53.8%) and 6 females (46.2%). Among the 362 patients studied, 6 patients (1.7%) had positive direct antibody test (DAT) or autocontrol results including 2 *β*-thalassemia patients (33%) and 6 SCD patients (67%) who developed autoantibodies. ABO and Rh phenotype frequencies among the patients in this study are presented in Table 3. The most common blood group was O (177 (49%)) followed by B (111 (30.8%)), A1 (65 (15.4%)), A2 (9 (3%)), and AB (9 (3%)). Prevalence of RhD+ was 88.1% while RhD- was 12.8%.

In the 34 *β*-thalassemia patients with alloimmunization, 57 alloantibodies were identified and are included in Table 4. Among these, anti-K alloantibody had the highest incidence being detected in 19/57 (33%) patients, followed by anti-E alloantibody (16/57 (28%) patients), anti-C antibody (8/57 (14%) patients), and anti-D and anti-C^w^ alloantibodies (both being detected in 4/57 (7%) patients). The least prevalent alloantibodies were anti-Jk^a^ (2/57 (3.5%) patients) and anti-c, and anti-S and anti-Fy^a^ alloantibodies (in 1/57 (1.8%) patients). Similarly, the prevalence of alloantibodies among *β*-thalassemia patients revealed that anti-K and anti-E alloantibodies had the highest incidence being detected in 6/13 (46%) and 5/13 (39%) patients, respectively (Table 4).

## 4. Discussion

RBC alloimmunization and autoimmunization are possible transfusion-related complications. While not all transfusion recipients develop alloantibodies or autoantibodies, RBC alloimmunization and autoimmunization can cause serious morbidity through delayed hemolytic transfusion reactions. In this study, RBC alloimmunization and autoimmunization frequencies were investigated in SCD and *β*-thalassemia patients in Al-Ahsa Region, Saudi Arabia.

Previous studies have reported varying rates of alloimmunization in Middle Eastern populations ranging from 12.98% to 39.42% in SCD patients and from 13.21% to 35.57% in *β*-thalassemia patients [16, 17, 21–23]. In the present study, alloimmunization rates were explored in SCD and *β*-thalassemia patients in Al-Ahsa Region, Saudi Arabia. The alloimmunization rate in chronically transfused *β*-thalassemia patients was 11.97%, similar to other Saudi studies [17]. However, Hindawi et al. revealed a higher frequency of alloantibodies (39.42%) in a similar study conducted in Jeddah City, Saudi Arabia. In the present study, the alloimmunization rate in SCD patients was 16.7% (Table 2), significantly lower than the rate reported by Hindawi et al. for SCD patients (35.75%).

The low rate of alloimmunization observed in SCD and *β*-thalassemia patients in the present study could be due to homogeneity of RBC antigens between blood donors and recipients [24]. In the King Fahad Hospital (KFH) in Al-Ahsa, most transfusion-dependent patients and donors are from Al-Ahsa Region. This homogeneity between local patients and donors may be one of the key factors contributing to the low prevalence of alloantibodies. Another possible explanation for the low alloimmunization rates in SCD and *β*-thalassemia patients is that antigen avoidance via phenotyping is employed at KFH for at-risk patients, including SCD and *β*-thalassemia patients, prior to RBC transfusions [25].

The present study was also designed to determine the most frequent alloantibodies among chronically transfused patients. In the 34 *β*-thalassemia patients with alloimmunization, 57 alloantibodies were found anti-K alloantibody (33%), anti-E antibody (28%), anti-C antibody (14%), and anti-D and anti-C^w^ antibodies (7%). The least prevalent alloantibodies were anti-Jk^a^ (3.5%), while anti-c, anti-S, and anti-Fy^a^ (1.8%) were the least detected antibodies. Similar to the prevalence of alloantibodies among *β*-thalassemia patients, anti-K (46%) and anti-E (39%) had the highest incidence in SCD patients (Table 4). The high frequencies of anti-K alloantibody and anti-E antibody are comparable to previous studies in Saudi Arabia, Egypt, Iran, and Michigan, USA [26].

In this study, the most common blood groups associated with alloimmunization in SCD patients were O RhD positive (41%) and B RhD positive (26%) (Table 4).

Overall, the autoimmunization rate in *β*-thalassemia patients in this study was 0.7%, significantly lower than rates reported by other studies [14, 15, 17, 21, 23]. The autoimmunization rate in SCD patients (5.3%) was higher than *β*-thalassemia patients, but significantly lower than rates reported by Halawani et al. (25%). Autoantibodies are frequently encountered during pretransfusion testing and can cause false positive results that may complicate serological compatibility testing to provide safe and compatible blood.

Sickle cell disease and thalassemia are highly prevalent in the Al-Ahsa Region of Saudi Arabia, leading to a large number of patients requiring multiple transfusions [6]. Alloimmunization is a common complication of transfusion therapy in these patients and can result in life-threatening hemolytic reactions. Our study provides important insights into the prevalence of red blood cell alloantibodies in this patient population and highlights the need for improved transfusion protocols to reduce the risk of alloimmunization. We recommend the use of extended phenotyping, including ABO, RH, Kell, Duffy, Kidd, and MNS blood group systems, in transfusion practice for patients with sickle cell disease and thalassemia in the Al-Ahsa Region. In addition, the implementation of a national registry for rare blood donor phenotypes could significantly benefit patients with sickle cell disease and thalassemia by reducing the risk of hemolytic transfusion reactions. Furthermore, the use of molecular genotyping, either alone or in combination with extended phenotyping, may provide a more accurate and comprehensive assessment of a patient's antigen profile, further minimizing the risk of alloimmunization and improving transfusion outcomes. Overall, our study highlights the need for continuous efforts to optimize transfusion protocols for patients with sickle cell disease and thalassemia in the Al-Ahsa Region to reduce the risk of alloimmunization and improve patient outcomes.

## 5. Conclusion

Blood transfusion-related alloimmunization and autoimmunization compromise the proper management of chronically transfused patients. This study was designed to determine the rates of alloimmunization and autoimmunization in SCD and *β*-thalassemia patients in Al-Ahsa Region, Saudi Arabia. Alloimmunization and autoimmunization are common among multiple transfused *β*-thalassemia patients in Saudi Arabia. The most frequent alloantibodies detected in this study were anti-K, anti-E, and anti-C antibodies. Ideally, extended phenotype matching should be implemented to prevent alloimmunization and reduce the risk of developing blood transfusion-related alloantibodies.

## Figures and Tables

**Table 1:** Sociodemographic characteristics of *β*-thalassemia and sickle cell disease patients in Al-Ahsa Region, Saudi Arabia.

	*β*-thalassemia		Sickle cell disease	
	Number	%	Number	%
*Sex*				
M	12	35	7	53.8
F	22	65	6	46.2
*Age*				
<17	4	12	2	17
17–24	9	26	3	17
25–45	20	59	7	50
46–60	1	3	1	17
>60	0	0	0	0
Total	34	11.97	13	16.7

**Table 2:** Rates of *β*-thalassemia and sickle cell disease alloimmunization in Al-Ahsa Region, Saudi Arabia.

	Current study	Jazan [17]	Jeddah [18, 19]	[20] Eastern
*β*-thalassemia alloimmunization (*n*)	34 out of 284	7 out of 53	27 out of 134	N/A
Percentage (%)	11.8%	13.21%	20.15%	N/A
*P* value		0.0010	0.0001	
Sickle cell alloimmunization (*n*)	13 out of 78	50 out of 385	30 out of 234	48 out of 350
Percentage (%)	16.7%	12.98%	12.8%	13.7%
*P* value		0.0002	0.0006	0.0010

**Table 3:** Blood groups of *β*-thalassemia and sickle cell disease patients in Al-Ahsa Region, Saudi Arabia.

Blood group	Number	%
A	65	15.4
A2	9	3
B	111	30.8
O	177	49
AB	9	3
RhD pos	327	88.1
RhD neg	47	12.8

**Table 4:** Alloantibodies present in *β*-thalassemia and sickle cell disease patients with alloimmunization in Al-Ahsa Region, Saudi Arabia.

		*β*-thalassemia		Sickle cell disease		Total	
System	Alloantibody	Number	%	Number	%	Number	%
RH	Anti-D	4	7	N/A	N/A	4	5.7
	Anti-C	8	14	1	7.7	9	12.9
	Anti-c	1	1.8	N/A	N/A	1	1.4
	Anti-E	16	28.1	5	38.5	21	30
	Anti-e	N/A	N/A	1	7.7	1	1.4
	C^w^	4	7	N/A	N/A	4	5.7

MNS	Anti-S	1	1.8	N/A	N/A	1	1.4

Lutheran	Anti-Lu^a^	1	1.8	N/A	N/A	1	1.4

Kell	Anti-K	19	33.3	6	46.2	25	35.7

Duffy	Anti-Fy^a^	1	1.8	N/A	N/A	1	1.4

Kidd	Anti-JK^a^	2	3.5	N/A	N/A	2	2.9

## Data Availability

The data that support the findings of this study are available from King Fahad Hospital-Al-Hofuf, but restrictions apply to the availability of these data, which were used under license for the current study, and so are not publicly available. Data are however available from the authors upon reasonable request and with permission of King Fahad Hospital-Al-Hofuf.
